# CONCERTO app for pediatric inpatients: A qualitative exploration of user experience and empowerment

**DOI:** 10.1371/journal.pone.0320924

**Published:** 2025-06-30

**Authors:** Hubert Rioux, Helena Bornet dit Vorgeat, Klara M. Posfay-Barbe, Frederic Ehrler, Nadia M. Bajwa

**Affiliations:** 1 Faculty of Medicine, University of Geneva, Geneva, Switzerland; 2 Innovation Center, Geneva University Hospitals, Geneva, Switzerland; 3 Department of General Pediatrics at the Children’s Hospital, Geneva University Hospitals in Geneva, Geneva, Switzerland; 4 Division of Medical Information Sciences, Geneva University Hospitals, Geneva, Switzerland; Queen's University Belfast, UNITED KINGDOM OF GREAT BRITAIN AND NORTHERN IRELAND

## Abstract

**Introduction:**

Hospitals increasingly use health information technologies such as websites and apps to foster patient engagement. The Geneva University Hospitals developed CONCERTO, an ecosystem of patient applications for this exact purpose. The objectives of this study were 1) to evaluate how pediatric patients and their parents use the app CONCERTO and 2) to pinpoint functionalities that could facilitate patient empowerment and consequently alleviate the stress of children and their parents related to hospitalization.

**Materials and methods:**

We interviewed 18 children and their parents during their hospitalization at the pediatric unit from May 20th to November 15^th^, 2022. Firstly, inspired by the Think-Aloud Protocol, we asked participants to complete a few specific tasks using the application. Then, we asked broader questions to better understand how CONCERTO could enhance patient empowerment. We used thematic analysis to explore the rich data set.

**Results:**

Children and their parents appreciated the features of the app. Specifically, they enjoyed creating an avatar and were grateful to have access to the interactive meal menu and the agenda. Both patients and parents would have appreciated learning about CONCERTO upon their arrival as it could have improved their hospital experience. Participants shared challenges they faced during their hospitalization and made suggestions about how more personalized content in CONCERTO could help overcome negative moments.

**Conclusions:**

CONCERTO effectively engaged and empowered patients and parents during their hospitalization. Further research is needed to evaluate the overall impact of such an app and understand the hurdles the care team faces in promoting the use of the app.

## Introduction

CONCERTO is a web app designed to enhance patient engagement during their stay at the Geneva University Hospitals (HUG). Both the pediatric and adult versions of the app were launched in 2019. CONCERTO gives access to a variety of personalized information and opportunities for social interaction. The goal is to provide patients with a sense of empowerment and to help them overcome the adversities of hospitalization. Table 1 provides an overview of the functionalities of the app Concerto.

**Table 1:** Overview of the functionalities included in CONCERTO and MyChart Bedside.

CONCERTO		MyChart Bedside^a^	
Functionalities	Brief description	Functionalities	Brief description
		*Home Screen*	Access to the medical record, hospital schedule, and medications
*MyAvatar*	Creation of an avatar		
		*Tutorial*	Access to material explaining how to use the app
*MyMenu*	Access to the meal menu and the possibility to personalize the meal	*Dining-on-Demand*	Access to food ordering
*MyAgenda*	Access to the patient’s hospital schedule	*Happening Soon*	Access to the patient’s hospital schedule
*MyTeam*	Identification (name, picture, and role) of the members of the patient’s care team	*Taking Care of Me*	Identification (name, picture, and role) of the members of the patient’s care team
*MyQuestions*	Notepad for the patient to write down notes, questions for the health care team	*Notes*	Personal notes, care team progress notes
		*I Would Like*	Online submission of personal requests to the care team
*MyAtlas*	Medical atlas with information about illnesses and medical procedures	*My Health*	Review of vitals and lab test results
		*To Learn*	Interactive learning materials (access to articles and videos)
*MyEntertainment*	Access to authorized online videos and games		
		*MyChart Signup*	Access to the outpatient portal

^a^Based on information provided by Gaughan and al. [1], Kelly and al. [2] and MyChart App Demo [3].

Self Determination Theory (SDT) underlies the frame and structure used to develop CONCERTO. SDT identifies three basic needs for optimal development: autonomy, competence, and relatedness [4]. Autonomy refers to one’s motivation, free of regulation from an external agent, such as rewards or a search for approval. Patients achieve competence if they receive the proper support and tools from the care team. Thus, relatedness, closely linked to autonomy and competence and represented by a patient-practitioner trust-based relationship, increases the patient’s likeliness to adopt healthy behaviours [5]. A meta-analysis of randomized controlled trials concluded that SDT has a small positive effect on promoting change for healthier behaviour [6]. In pediatric care, SDT helps foster self-management of chronic health conditions among adolescents and emerging adults [7], and eases their transition to adult healthcare [8].

Concerns and fears of hospitalized children may include: separation from family and friends, being in an unfamiliar environment, undergoing investigations and treatments, and loss of self-determination [9]. Many other authors have described the pain, anxiety, and fear that children and parents feel in relation to their hospitalization [10,11]. The hospital environment can lead to pediatric medical traumatic stress [12,13], defined by the US-based National Child Traumatic Stress Network as “a set of psychological and physiological responses of children and their families to pain, injury, serious illness, medical procedures, and invasive or frightening treatment experiences. Medical trauma may occur as a response to single or multiple medical events” [14].

Better communication between the care team and the child is required to help overcome hospitalization-induced stress. Coyne advocates that “hospital environments need to be more child-centered. It is essential that children’s views be elicited in the delivery of their care” [9]. Accordingly, some hospitals have developed activities to enhance children’s experiences. Recreational play seems to improve positive affect and lessen sadness of patients [15]. Patient’s stress may decrease, and self-determination may increase by maintaining contact with outside friends, family, and school [9]. Some authors noted that using technology such as virtual reality could increase motivation towards treatment [16] and reduce pain and anxiety reported by patients before or during medical procedures [17]. Videoconferencing with relatives during hospitalization can also reduce stress among children and their families [18].

In order to foster patient engagement, hospitals are increasingly using health information technologies through the development of websites and apps. In Europe and North America, apps such as CONCERTO have been included in pediatric services, but few studies have evaluated their impact. Some institutions, like the HUG have developed their own app, while others, mainly in North America, have chosen to integrate the existing inpatient portal in MyChart Bedside developed by Epic (an overview of its functionalities can be found in Table 1). A portal such as MyChart Bedside was seen as a way to better understand medications and communicate with nurses [19]. One study concluded that parents felt that MyChart Bedside improved their understanding of their child’s condition and evolution, which helped them make care decisions [20]. However, the longer the hospitalization, the less likely a patient is prone to use the portal [21].

Patient portals are considered effective tools for increasing patient engagement by empowering patients to partner in their healthcare, but further research is needed [22]. Ultimately, a better understanding of the user experience is needed to improve such patient portals’ design, implementation, and benefits [23,24]. The objective of our study is two-fold. First, to evaluate how patients use the app CONCERTO. Second, to pinpoint features that could facilitate patient empowerment and alleviate the stress of children and their parents related to hospitalization.

## Materials and methods

For the reporting of this study, we applied the Standards for Reporting Qualitative Research as formulated by O’Brien and al. [25].

### Qualitative approach

To explore our research questions, we were guided by the grounded theory principles of emergence, theoretical sampling, constant comparison, and theoretical saturation [26–28]. Throughout the study, we remained open to identifying new perspectives based on our patients’ and their parents’ experiences and realities, allowing theory to emerge from the data. To create a rich data set, we sought participants who represented a diversity of ages, genders, and experiences with different illnesses in our chosen setting, and theoretical sampling guided participant selection as emerging concepts were further explored. We employed the iterative constant comparison method, to structure our coding method and to inform our thematic analysis, comparing new data with previously coded data to refine categories and ensure they were grounded in participants’ experiences. The continuous literature review on patient empowerment and hospitalization stress complemented this process without influencing the emergence of themes. HR discontinued the interviews when no new themes were identified by HR and NB during the thematic analysis. Coding discrepancies were resolved through team discussions. The study process is further described in detail below.

### Development of the interview guide

HR and NB developed the interview guide. HR is a 5th-year medical student with a journalist background and conducted the interviews. NB is a pediatric residency program director with a medical education background.

Each interview was composed of two parts. Firstly, we asked participants to complete a few specific tasks and describe what they were doing along the way, so that we would better understand how patients are using CONCERTO. To do so, we were inspired by the Think-Aloud Protocol, which has been used in a number of studies to assess user experience with technology, notably for evaluating the inpatient portal MyChart Bedside [24].

The user experience then served as a basis for the second part of the interview. Each interview was recorded, transcribed, and coded. Guiding concepts emerged from the first interviews and contributed to further tailoring of questions for the next interviewees. HR and NB fine-tuned the questions throughout the process until no new themes were identified. The initial interview guide can be found in S1 File found in the Supporting information.

### Ethical approval

This study was submitted to the Geneva Research Ethics Commission (Req-2022–00409) and received an exemption from review. The Research Ethics Commission stated that the study protocol respected general ethical principles. Parents of participants provided written informed consent and pediatric patients provided assent.

### Patient recruitment and data analysis

We interviewed selected children and their parents during their stay at a HUG pediatric unit from from May 20th to November 15^th^, 2022. This hospital unit has a supervising nurse and a nurse responsible for CONCERTO. All participants received an information sheet detailing the study procedure and the nature of their involvement and a 30 CHF gift certificate to a book and electronics store. Children gave verbal assent, and parents signed a written informed consent form before beginning participation.

We aimed to interview children hospitalized for acute or chronic care, boys and girls from 2 to 16 years of age, and their parents. Before going to the pediatric unit to conduct an interview, HR called the nursing team and asked if there were potential participants, namely French speakers with parents present during the hospitalization. HR would then meet the preselected candidates to explain the scope of the study, the study procedure, and to find out if they were interested in participating. The interested candidates were then given an iPad with access to the app CONCERTO and had at least 30 minutes to navigate it before HR returned to conduct the interview.

The interviews were conducted in French and audio recorded. For transcription, we used the *dictate* function from Microsoft Word. The transcripts were then compared with the recordings and reviewed before analysis and coding. The transcribed interviews were anonymized to protect patient confidentiality. To analyze the verbatim, NB and HR met regularly to create the code book and to categorize themes. After the first round of six interviews, HR conducted 12 additional interviews until no new themes were identified by HR and NB (18 interviews total). After the initial open coding, axial coding allowed us to refine relationships between categories, leading to three main concepts: patient’s view on hospitalization, patient’s experience with CONCERTO, and patient empowerment*.* Coding discrepancies were resolved through team discussions held during the coding process.

Two of the authors (NB, HR) translated pertinent citations to English at the writing stage, keeping in mind that the source language should not be misconstrued [29]. The translation procedure described by Chen and Boore [30] was followed and the authors used back-translation in search of better translation accuracy.

## Results

In total, we conducted 18 interviews between May 20^th^, 2022 and November 15^th^, 2022. Boys and girls were equally represented. The age average was 10 1/2 years old. We specifically categorized the children into three age groups (2 to 6, 7 to 11, and 12 to 16). Overall, four children from 2 to 6 years of age, five from 7 to 11, eight from 13 to 16, and one child was 17. Four interviews conducted with children 12 years and older were held without the direct presence of their parents. The younger the child’s age, the more a parent would play a central part in the interview.

Throughout the results, we cited relevant comments and identified whether a parent made them [P] or a child [C]. We also specified the age of the child, e.g., a comment made by a 10-year-old participant is marked [C-10]. A summary of the quotes can be found in Table 2.

**Table 2:** Quotes translated from French to English.

App function	Quotes from children	Quotes from parents
**Log-in**		“It was not complicated at all, quite user-friendly.” “I’m not concerned about the time, but I just want to make sure that they don’t get access to other stuff.” *“It should be systematic; we should know about it when we come in. Somebody could come and take 10 minutes to tell us about the app, just like you did”*
**Graphic design**	“The graphic design is so good! Visually, it is so pretty, which I think is important. It’s nice because it helps makes things easier to understand” [15 y.o.]	
**MyAvatar**	“I think it is nice. It gives me something to do. Creating an avatar is so cool!” [15 y.o.]	“The shape of the mouth, the eyes, and the accessories… we laughed a lot while playing with those ones...He created a cat policeman. It’s pretty good; it’s creative.”
**MyMenu**	“This was the best surprise… Now, I know what I’ll be eating tonight, and I love meatballs and spaghetti! I am very happy!” [15 y.o.] “let’s say I see a burger with fries on the meal menu; it will stress me out for the whole day. So, it depends on the illnesses or the reasons why people are hospitalized” [13 y.o.]	*“It can help us not to ask useless questions to the medical staff. Not useless, but I mean not health-related questions. So now we know the meal menu in advance”* *“We were told about it late after our admission. At one point, they saw that my child wouldn’t eat the meals. That’s the moment they told us about it. I think it’s too bad because we would have used it right upon our arrival”*
**MyAgenda**	“It’s very well designed. For example, I had appointments that I had forgotten about. I checked, and I told myself that tomorrow I had a hypnosis session” [15 y.o.] *“I feel a little stressed with the needles and all… I just want to go home”* [17 y.o.]	“I like the agenda where we see our appointments and treatments for the day. It’s nice to just know what’s coming up with an approximate timeline. It’s life-changing!”
**MyTeam**	“Since there are the pictures of the medical staff, I think there should be the ones of the creative and education teams” [14 y.o.]	“It’s nice because we too often forget names.” *“I would have appreciated having psychological support earlier, the intervention of the child psychiatrists. I already shared that thought because it was hard for me, for my husband too.”* *“We should not overdo it. Human contact is very important”*
**MyQuestions**	“I can now write down my questions, so at least I don’t forget them. Because every time I have a treatment or appointment, there are questions that I forget to ask” [15 y.o.]	“Sometimes the doctors come for a short visit, and they give us a lot of information. Once they are gone, that’s the moment when thousands of questions come to our minds. So, we then have to wait until the next day to ask them, and we sometimes forget.” “Sometimes I ask questions, and I have the feeling that I bother the medical staff and that they have other things to do. It’s difficult afterward to ask for other things. We want to better understand, but speaking with somebody who seems to be in a rush, it’s never pleasant. So it could be good to have a tool to ask questions directly to the doctor who could answer when the time comes”. *“If we have a moment of doubt once we have left the hospital, we could ask questions and have someone designated to answer. It does happen quite often that we are left with unanswered questions back home. e”* *What I find it difficult not to know. In some ways, we wish for a diagnosis, but nothing comes up. And at the same time, we don’t wish to find anything. So, it is a bit complicated. Not knowing if it will stop someday or carry on. Will it end well? Will it end badly?“*
**MyAtlas**	“it helps to quickly find answers to our inquiries. Plus, it is a reliable source of information” [13 y.o.]	“Being able to know more about our illness or of others’, I saw that there are summaries of many illnesses, reasons why people are hospitalized… We can learn an lot.” “it would be nice to offer a choice menu with the first three typed letters. Nothing is offered; for example, if I write down DIA, nothing comes up. Other search tools would sometimes offer DIABETES.” “It is not pediatric-oriented content. It is a concern for me that my child could access medical details about adult-related pathologies.”
**MyEntertainment**	“I like the app because I can entertain myself, I don’t just lie down in my bed doing nothing” [10 y.o.] “it could provide games, animated films, podcasts, or even a virtual magazine with event listings. It could also provide music, reading suggestions, things like that” [13 y.o.] *“Compared to home, I’m using my cell phone a lot more”* [12 y.o.]	“I think it lacks games and videos for all ages” *“I think we need to be cautious. We have to use tablets in moderation”*

Patient empowerment quotes are in italics.

### Patient’s view on hospitalization

During the interview, we asked participants how they felt about their hospitalization and what could be done when facing an unpleasant experience. Parents and children alike expressed how challenging a hospitalization can be. Despite these challenges, participants were very grateful for the care team’s work. Some had a sense of self-sacrifice while going through the medical procedures.

Of the more negative experiences, many children told us that they were just plain bored or felt sad; others talked about sleeping difficulties and being scared or were apprehensive about having to do blood samples: “I feel a little stressed with the needles and all… I just want to go home” [C-17]. Many parents felt insecure and powerless because of the uncertainties linked to the diagnosis or the upcoming medical procedures: “What I find difficult is not knowing. In some ways, we wish for a diagnosis, but nothing comes up. And at the same time, we don’t wish to find anything. So, it is a bit complicated. Not knowing if it will stop someday or carry on. Will it end well? Will it end badly?” [P]. Some parents feared the long-term impact of the hospitalization on their child and the family and felt that more psychological support should be available.

### Patient’s experience with CONCERTO

#### Log-in.

Overall, participants had no difficulties logging into the app: “It was not complicated at all, quite user-friendly” [P]. After scanning or entering the patient’s HUG identity number, an authentication code is sent to a cell phone number registered in the patient’s medical file, most likely one of the parents. Many users appreciated the security associated with this authentication code. Although, access sometimes proved to be tricky due to the authentication code. For some users, such a code was a limiting factor in logging into the app, as the phone number indicated in the patient’s medical file was not the one associated with the patient’s parent device. This obstacle limited patients from using CONCERTO and participating in the study. Parental control concerning access to other sites available on the patient’s device also worried some parents.

#### Graphic design.

Once logged into the app, the graphic design was overwhelmingly appreciated and considered an important asset for children: “The graphic design is so good! Visually, it is so pretty, which I think is important. It’s nice because it helps makes things easier to understand” [C-15]. Some children felt that a video tutorial would be useful to show all functions available, with maybe a character guiding the youngest ones. The children unable to read found it harder to play with the app; one parent suggested that having separate child and parent versions would be useful.

#### MyAvatar.

Creating an avatar was a big hit for the vast majority of the children. It was seen as a fun way to pass some time and express creativity: “I think it is nice. It gives me something to do. Creating an avatar is so cool!” [C-15]. Parents also enjoyed the function. Some children expressed the desire to have more options, like a greater variety of t-shirt designs.

#### My Menu.

The interactive meal menu and the avatar were among the two most appreciated functions. Patients were relieved to finally know what they would eat and that they could ask for some changes or do it directly through the app: “This was the best surprise… Now, I know what I’ll be eating tonight, and I love meatballs and spaghetti! I am very happy!” [C-15]. Some parents also thought it was practical. Although for some children with eating disorders, the meal menu could provoke anxiety: “Let’s say I see a burger with fries on the meal menu; it will stress me out for the whole day. So, it depends on the illnesses or the reasons why people are hospitalized” [C-13].

#### MyAgenda.

For most parents, the agenda was seen as a huge asset by providing information on upcoming treatments and medical visits. Parents said they appreciated knowing those details in order to better plan the daily family logistics: “I like the agenda where we see our appointments and treatments for the day. It’s nice to just know what’s coming up with an approximate timeline. It’s life-changing!” [P]. Older children also liked knowing their schedule in advance. Some users thought more details should be provided, like the name of the caregiver or a reminder of an upcoming event.

#### MyTeam.

As for fostering communication, having the names, pictures, and roles of the care team members is fulfilling a need: “It’s nice because we too often forget names” [P]. Participants felt that the pictures of all other collaborators (e.g., music teachers, hospital clowns, etc.) should be available online.

#### MyQuestions.

MyQuestions was considered a useful way to write down questions to be asked and not to be forgotten. “I can now write down my questions, so at least I don’t forget them. Because every time I have a treatment or appointment, there are questions that I forget to ask” [C-15]. For some, it could be a way to communicate more efficiently with the medical staff. It could even be a communication tool after the hospital discharge.

#### MyAtlas.

Many children felt that the health atlas had interesting content: “It allows us to quickly find answers to the subjects that interest us. And we are lucky that it is a reliable source of information” [C-13]. Other users felt that it was difficult to search and find relevant information about one’s condition: “It would be nice to offer a choice menu with the first three typed letters. Nothing is offered; for example, if I write down DIA, nothing comes up. Other search tools would sometimes offer DIABETES” [P]. Some parents thought some health-related content was not well-suited for younger children: “It is not pediatric-oriented content. It is a concern for me that my child could access medical details about adult-related pathologies” [P].

#### MyEntertainment.

For some children, access to entertainment (e.g., movies, video games) is crucial: “I like the app because I can entertain myself, I don’t just lie down in my bed doing nothing” [C-10]. Most felt the app did not offer enough games, videos, or podcasts. Parents and teenagers felt that content should be more age specific.

#### Patient empowerment.

Throughout the interviews, participants expressed some negative feelings related to their present or past hospital stays. They affirmed that an app like CONCERTO could help them to feel more engaged during their hospitalization. The quotes in italic found in Table 2 relate to these testimonies.

Based on the interviews, the app gives patients and their parents a better sense of empowerment. To both parents and patients, CONCERTO allowed them to find basic information such as their menu and agenda while at the same time allowing them to solicit the care team for more pressing questions: “It can help us not to ask useless questions to the medical staff. Not useless, but I mean not health-related questions. So now we know the meal menu in advance” [P]. Just having something to play with (e.g., creating an avatar) also gave them a way to escape the harsh reality of a hospital bed.

Before introducing ourselves and the purpose of this research, few patients and parents had heard of the app CONCERTO: “We were told about it late after our admission. At one point, they saw that my child wouldn’t eat the meals. That’s the moment they told us about it. I think it’s too bad because we would have used it right upon our arrival” [P]. A parent deplored that the app was not systematically advertised to newcomers: “It should be systematic; we should know about it when we come in. Somebody could come and take 10 minutes to tell us about the app, just like you did” [P]. Therefore, participants would most likely have spent little time using the app before answering our questions. Nonetheless, patients seemed enthusiastic about using it for the remainder of their stay.

However, participants were doubtful about how an app could help them overcome negative feelings felt during their hospitalization. A more personalized portal with facilitated access to medical files (e.g., electronic health records) and related content (customized health atlas) was perceived as a way to overcome that sensation of being left out. Parents shared several ideas on how to improve their stays, such as parking notifications, a mapping of the unit with information leading to the closest shower, and a complaint board. Participants suggested more online entertainment, serious games for surmounting needle anxiety, relaxation videos, self-hypnosis, and promotion of sports activities.

On the other hand, some participants wished to prevent too much technology intrusion: “We should not overdo it. Human contact is very important” [P]. Some were also seeking more freedom of movement and access to activities other than digital. Socio-cultural activities and outside playgrounds are already offered at the HUG, and CONCERTO could promote them more actively.

Some worries have been expressed over the screen time associated with using the app. Many children already had their own devices and were already killing time on social media and online gaming. One of them told us: “Compared to home, I’m using my cell phone a lot more” [C-12]. For some parents, control over screen time would be welcomed: “I think we need to be cautious; we have to use tablets in moderation” [P].

## Discussion

These interviews gave us a better understanding of the most appreciated features of CONCERTO and allowed us to identify where improvement is needed. Features like the interactive MyMenu are an engaging way to give families a sense of empowerment, but other functions like a personalized health atlas or parking notifications would need substantial investment to achieve similar goals. The importance of a communication strategy to increase the uptake of CONCERTO was a recurring theme in our study.

The testimonies of children and parents seem aligned with previous studies on patient portals and their potential to enhance a patient’s participation during a hospitalization [19,20]. Some participants expressed how hospitalization can have negative impacts, mirroring previous studies on the subject [12,31,32]. CONCERTO does seem to have potential benefits to alleviate some of these situations, but further research is needed to fully understand how effective it can be, as other studies concluded [22–24,33].

CONCERTO has been described by participants as an additional tool to foster communication with the care team, like other similar apps [2,19]. However, the low level of implication of the health care team in promoting CONCERTO reflects a previous survey on the difficulties of implementing the use of the portal MyChart Bedside, and that better training seems needed [34].

The main limitation of our investigation is due to the small amount of time the patients and their parents spent using the app before answering our questions. If all patients were introduced to the app upon their arrival, we could have recruited patients who had already spent a few days in the pediatric unit. This would have provided a richer insight into how such an app could enhance patients’ empowerment. We, therefore, lack some foresight to conclude if the app would be used regularly or if patients would use it less as the hospitalization goes on, as another study concluded [21]. An age-specific structured interview would have also helped to foster interactions with the younger participants. The younger the patient was, the more a parent would answer the questions alone, their children playing mainly with MyAvatar and not being interested in other functions. Translation of some of the quotes from French to English was also limited by the use of slang by our participants. Future studies addressing these concerns may lead to a new theory on the intersection of the use of health technology, patient empowerment, and stress related to hospitalization.

A better understanding of how the care team could help implement the use of CONCERTO would be essential. Future studies are needed to include the perspectives of the care team in implementing CONCERTO. Similar challenges have also been raised by the inpatient portal MyChart Bedside [1].

## Conclusions

Most patients and their parents considered CONCERTO effective in engaging them during their hospitalization. In accordance with other studies, further research is needed to evaluate the overall impact of such an app and to better understand the hurdles faced by the care team that prevent them from promoting the application.

## Supporting information

S1 File Appendix. Structure of the interviews. (DOCX)
