# Supplementary material for: CONCERTO app for pediatric inpatients: A qualitative exploration of user experience and empowerment
Source: PLoS One. 2025 Jun 30;20(6):e0320924. doi: 10.1371/journal.pone.0320924 (PMC12208447; doi:10.1371/journal.pone.0320924)
Supplement: S1 File — (DOCX) [file pone.0320924.s001.docx]

**S1 APPENDIX – Structure of the interviews**

**Part 1 – Navigating through CONCERTO – 15-30 minutes**

Let's take a moment to play with the app CONCERTO. Please talk to me while you are using it: tell me what you are doing, if you find any difficulties, or what you enjoy about the app.

1. Can you find the app and open it for me?

2. Could you create an avatar that looks like you?

3. Could you tell me who is taking care of you today?

4. Do you know where to find your planning for the day?

5. Let's say you feel hungry. Can you tell me what is the food menu today? Can you figure out how to choose your menu?

6. Can you note a question for your doctors or your nurses?

7. If you needed to find information about your medical unit, where would you look?

8. Can you tell me why you are in the hospital? Could you find more information about your condition?

**Part 2 – explanatory data collection – 30-45 minutes**

Initial research question: in what way can CONCERTO help to alleviate the stress of children and their parents related to hospitalization?

1. Please describe what CONCERTO is in your own words.

2. What do you think of the app CONCERTO?

3. How does CONCERTO fit into your experience at the HUG?

4. Please tell me about your current hospitalization.

5. How have you been feeling during your hospitalization?

6. Have you had negative experiences during this hospitalization? Or a previous one? Please explain.

7. How could an app like CONCERTO be useful to you while being hospitalized?

8. How could it help you overcome difficulties met during your hospitalization?

9. Do you feel that CONCERTO helps you to communicate with the care team?

10. Do you feel that CONCERTO is easy to use? Would you need more information about how to use the app?

11. Would you like to add any information or comments about what we have discussed?
